# Use of lectins to in situ visualize glycoconjugates of extracellular polymeric substances in acidophilic archaeal biofilms

**DOI:** 10.1111/1751-7915.12188

**Published:** 2014-12-09

**Authors:** R Y Zhang, T R Neu, S Bellenberg, U Kuhlicke, W Sand, M Vera

**Affiliations:** 1 Aquatische Biotechnologie, Biofilm Centre, Universität Duisburg – EssenUniversitätsstraße 5, 45141, Essen, Germany; 2 Department of River Ecology, Helmholtz Centre for Environmental Research-UFZBrueckstrasse 3A, 39114, Magdeburg, Germany

## Abstract

Biofilm formation and the production of extracellular polymeric substances (EPS) by meso- and thermoacidophilic metal-oxidizing archaea on relevant substrates have been studied to a limited extent. In order to investigate glycoconjugates, a major part of the EPS, during biofilm formation/bioleaching by archaea on pyrite, a screening with 75 commercially available lectins by fluorescence lectin-binding analysis (FLBA) has been performed. Three representative archaeal species, *Ferroplasma acidiphilum* DSM 28986, *Sulfolobus metallicus* DSM 6482^T^ and a novel isolate *Acidianus* sp. DSM 29099 were used. In addition, *Acidianus* sp. DSM 29099 biofilms on elemental sulfur were studied. The results of FLBA indicate (i) 22 lectins bound to archaeal biofilms on pyrite and 21 lectins were binding to *Acidianus* sp. DSM 29099 biofilms on elemental sulfur; (ii) major binding patterns, e.g. tightly bound EPS and loosely bound EPS, were detected on both substrates; (iii) the three archaeal species produced various EPS glycoconjugates on pyrite surfaces. Additionally, the substratum induced different EPS glycoconjugates and biofilm structures of cells of *Acidianus* sp. DSM 29099. Our data provide new insights into interactions between acidophilic archaea on relevant surfaces and also indicate that FLBA is a valuable tool for in situ investigations on archaeal biofilms.

## Introduction

Microbial leaching of metal sulfides (MS) is an expanding biotechnology (Brierley and Brierley, 2013). However, it can also occur as an unwanted natural process called acid rock drainage or acid mine drainage (AMD). This process is accompanied by acidification and heavy metal pollution of water bodies and can cause serious environmental problems (Kalin *et al*., 2006; Sand *et al*., 2007). Acidophilic archaea including genera such as *Ferroplasma*, *Acidianus*, *Sulfolobus* and *Metallosphera* play important roles in bioleaching and AMD systems, and have received significant attention for commercial applications (Olson *et al*., 2003; Golyshina and Timmis, 2005; Rawlings and Johnson, 2007).

The genera *Acidianus* and *Sulfolobus* are thermoacidophiles found in hydrothermal vents or bioleaching systems at temperatures above 60°C. They are capable of oxidizing both iron(II) ions and reduced inorganic sulfur compounds (RISCs). Biological ferric iron regeneration and acidic conditions are crucial for the dissolution of MS (Schippers and Sand, 1999; Sand *et al*., 2001). Under thermophilic conditions, iron oxidation is accelerated, and the passivation of chalcopyrite (CuFeS_2_) surfaces by RISCs is nearly eliminated, which has significant importance in the biomining industry.

The mesophilic archaeon *Ferroplasma acidiphilum* was first isolated from a semi-industrial bioleaching reactor processing arsenopyrite in Kazakhstan (Golyshina *et al*., 2000). It oxidizes iron(II) ions or pyrite in the presence of trace amounts of yeast extract. In addition, all isolated strains of *Ferroplasma* spp. can grow heterotrophically (Dopson *et al*., 2004). *Ferroplasma* is frequently detected in biomining ecosystems and is considered to be a major player in global iron and sulfur cycles in highly acidic environments (Edwards *et al*., 2000; Golyshina and Timmis, 2005; Chen *et al*., 2014).

Biofilms are defined as interface-associated communities of microorganisms embedded in extracellular polymeric substances (EPS). The EPS usually consist of polysaccharides, proteins, lipids and DNA. They are generally subdivided into two types: ‘capsular EPS’ are tightly bound to cells, while ‘colloidal EPS’ are loosely bound to cells and can be easily released (e.g. by centrifugation or washing) into the solution (Nielsen and Jahn, 1999). EPS are essential for biofilm structure and function due to their involvement in cellular associations, nutrition exchange and interactions of microorganisms with their bio-physicochemical environment (Wolfaardt *et al*., 1999; Neu and Lawrence, 2009). EPS are also involved in the attachment and biofilm formation of leaching microorganisms to surfaces of MS, which is an essential step at the start of the leaching process (Vera *et al*., 2013). Biofilms formed by heterotrophic prokaryotes or phototrophs are usually dynamic structures that can grow to thick three-dimensional macro-communities (Stoodley *et al*., 2002). In contrast, the majority of metal-oxidizing microorganisms attach directly to the surface of MS, forming monolayer biofilms. By this lifestyle, cells can obtain energy from Iron(II) ions or RISCs, which are released during the dissolution of the MS. Interestingly, two distinct biofilm morphologies were described for *Ferroplasma acidarmanus* Fer1: A multilayer film was formed on pyrite surfaces after 38 days of incubation, and up to 5 mm-long filaments were found on sintered glass spargers in gas lift bioreactors (Baker-Austin *et al*., 2010).

Few studies have shown biofilms of archaea, including thermoacidophiles, halophiles and methanogens. The first archaeal biofilm was described for the hyperthermophilic *Thermococcus litoralis*, which developed in rich media on polycarbonate filters and glass surfaces (Rinker and Kelly, 1996). *Pyrococcus furiosus* and *Methanobacter thermoautotrophicus* developed monospecies biofilms on solid surfaces (Näther *et al*., 2006; Thoma *et al*., 2008). Bi-species biofilm development of *P. furiosus* and *Methanopyrus kandlerii* was shown to be established within less than 24 h on abiotic surfaces (Schopf *et al*., 2008). Biofilm analysis of three *Sulfolobus* spp. showed that their structures were different, ranging from simple carpet-like structures in *Sulfolobus solfataricus* and *Sulfolobus tokodaii* to high density tower-like structures in *Sulfolobus acidocaldarius* in static systems. All three species produced EPS containing glucose, galactose, mannose and N-acetylglucosamine (GlcNAc) once biofilm formation was initiated (Koerdt *et al*., 2010). Biofilm formation by methanogenic archaea under static conditions was studied by confocal laser scanning microscopy (CLSM) and scanning electron microscopy. The three species, *Methanosphaera stadtmanae*, *Methanobrevibacter smithii* and *Methanosarcina mazei* strain Gö1, formed mainly bilayer biofilms on mica surfaces. Nevertheless, the development of multilayer biofilms was also observed (Bang *et al*., 2014). Biofilm formation of haloarchaea, including species of *Halobacterium*, *Haloferax* and *Halorubrum*, was investigated by a fluorescence-based live cell adhesion assay. Cellular appendages were speculated to be involved in the initial attachment (Fröls *et al*., 2012). Two types of biofilm structures were detected including carpet-like multilayers and large aggregates adhering to glass surfaces. Similar as occurring in the acidophilic archaea such as *Sulfolobus* and *Ferroplasma* (Baker-Austin *et al*., 2010; Koerdt *et al*., 2010; Zhang *et al*., 2014), biofilm development occurs in a surprisingly wide variety in haloarchaea. In addition, EPS like eDNA and various glycoconjugates were found to be present in these biofilms (Fröls *et al*., 2012).

Lectins are proteins or glycoproteins capable of binding reversibly and specifically to carbohydrates without altering their structures. Fluorescence lectin-binding analysis (FLBA) represents the only option for non-destructive and in situ glycoconjugate analysis and, therefore, is widely used in glycoconjugate/biofilm analysis in combination with other fluorochromes, e.g. specific for nucleic acids (Zippel and Neu, 2011; Bennke *et al*., 2013; Castro *et al*., 2014). Furthermore, their multivalency ensures high-affinity binding to the cell surface and biofilm structures containing various glycoconjugates. Only a few lectins combined with nucleic acid dyes have been used in investigations on acidophilic biofilms related to bioleaching and AMD systems. The most frequently used lectin is Concanavalin A (Con A) from the jack-bean, *Canavalia ensiformis*, binding to mannose and glucose residues (Goldstein *et al*., 1965). Con A has been used to visualize various acidophilic archaeal and bacterial biofilm cells, e.g. *Sulfolobus* (Koerdt *et al*., 2010; Zolghadr *et al*., 2010; Bellenberg *et al*., 2012), *F. acidiphilum* (Zhang *et al*., 2014) and *Metallosphaera hakonensis* (Africa *et al*., 2013). As EPS are complex mixtures consisting of many types of macromolecules, it is impossible to address their complexity with a single staining approach. Even for the similar glycoconjugates, multiple lectin probes have to be used (Neu and Lawrence, 2009). Thus, it is necessary to screen a library of lectins in order to find the ones binding to the glycoconjugates in a particular biofilm (Peltola *et al*., 2008; Zippel and Neu, 2011; Bennke *et al*., 2013).

To date, EPS production and biofilms of archaeal species have been investigated only to a limited extent, especially concerning the ones growing in acidic environments (Orell *et al*., 2013). Nevertheless, it is essential to visualize EPS glycoconjugate identity and distribution on relevant surfaces together with analysis of their chemical composition to understand their function(s) in bioleaching. In the present study, three representative archaeal strains – a euryarchaeote *F. acidiphilum* DSM 28986 and two crenarchaeota, *Sulfolobus metallicus* DSM 6482^T^ and *Acidianus* sp. DSM 29099 – were selected for FLBA of their EPS glycoconjugates and biofilm structures during bioleaching of pyrite as well as on elemental sulfur in case of *Acidianus* sp. DSM 29099. In order to image EPS glycoconjugates in these biofilms, 75 commercially available lectins were tested for applicability. This is the first report of EPS glycoconjugate probing by means of FLBA for archaeal biofilms in situ during bioleaching.

## Results and discussion

### Visualization of attached archaea and biofilms

In previous reports, acridine orange (Fröls *et al*., 2012), fluorescein (Baker-Austin *et al*., 2010) and DAPI (Henche *et al*., 2012; Koerdt *et al*., 2012) have been used for staining acidophilic archaeal species. In order to display the distribution of archaeal cells as well as to visualize EPS including proteins, nucleic acids and lipophilic compounds in biofilms on pyrite surfaces, six fluorochromes including SybrGreen (Invitrogen, Carlsbad, CA, USA), Syto 9 (Invitrogen), Syto 64 (Invitrogen), SyproRed (Invitrogen), SyproOrange (Invitrogen) and FM4-64 (Invitrogen) were selected to evaluate their potential suitability (Table 1). Sypro stains like SyproRed and SyproOrange were originally developed for measuring protein concentrations in solution or in gels. Later, they were used for flow cytometry studies (Zubkov *et al*., 1999) and finally for staining the biofilm matrix for CLSM examination (Neu and Lawrence, 1999a; Lawrence *et al*., 2003). FM-dyes (FM4-64 and FM1-43) are widely used to study endocytosis, vesicle trafficking and organelle organization in living eukaryotic cells (Bolte *et al*., 2004).

**Table 1:** List of dyes and their Ex and Em wavelengths and associated binding targets

Dyes	Specificity	Ex/Em wavelength (nm)	Company
SYTO 9	NA	483/478–488, 500–560	Invitrogen
SYTO 64	NA	483, 599/475–489, 625–700	Invitrogen
SybrGreen	NA	483/475–489, 500–560	Invitrogen
FM4-64	Lipid-rich domain	483, 506/650–790	Invitrogen
SyproRed	Proteins	475, 500/470–480, 580–680	Invitrogen
SyproOrange	Proteins	475/470–480, 520–620	Invitrogen
TRITC or Alexa 488-conjugated lectins	EPS glycoconjugates	490/505–545	EY Laboratories, Inc.
FITC-conjugated lectins	EPS glycoconjugates	490/485–495, 510–600	Sanbio Laboratory/EY Laboratories, Inc.

Em, emission; Ex, excitation; FITC, fluorescein isothiocyanate; NA, nucleic acids; TRITC, tetramethyl rhodamine isothiocyanate.

#### Archaeal cells and biofilms on pyrite

**Fig 1:**
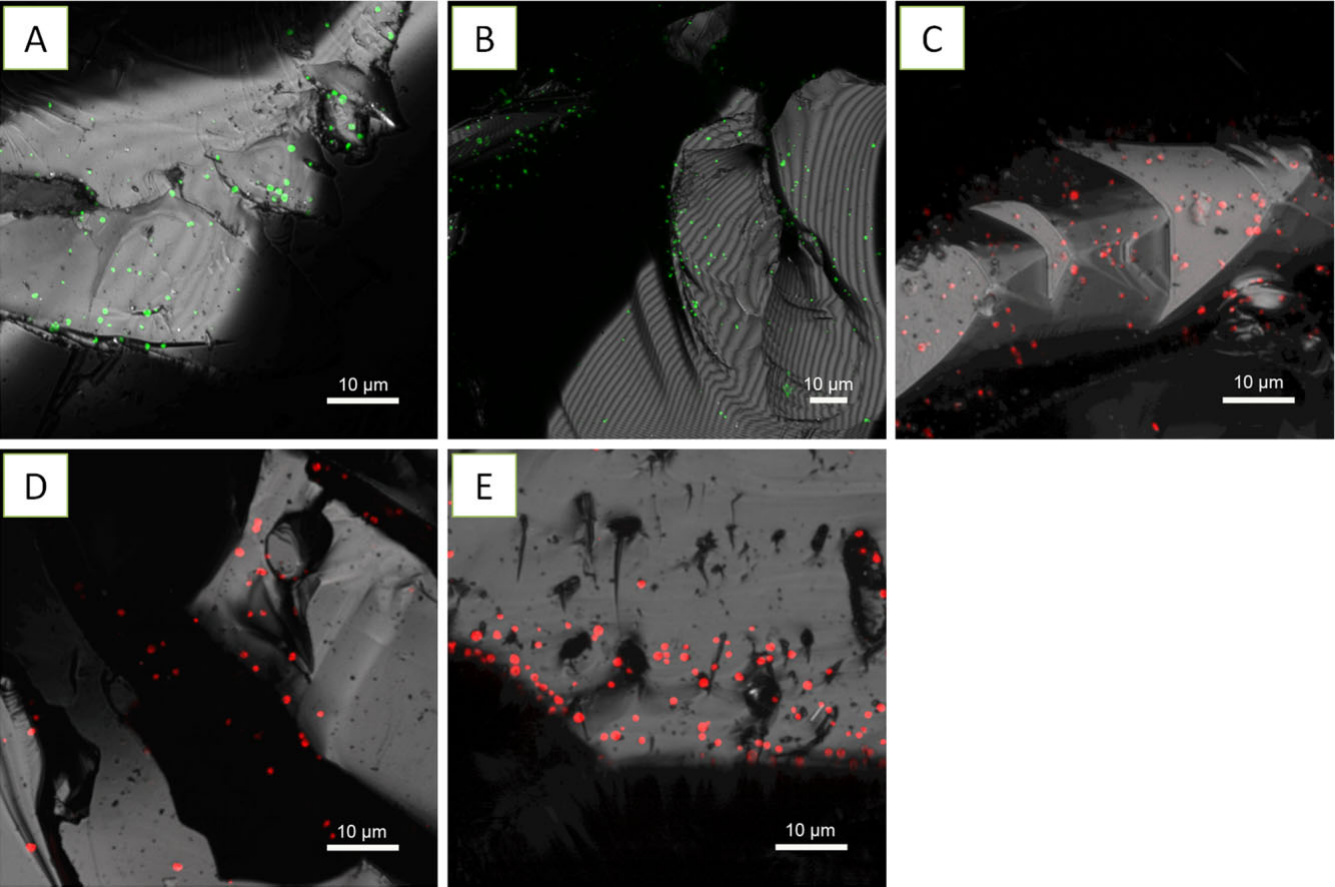
Maximum intensity projections of *F. acidiphilum* DSM 28986 biofilms on pyrite stained by SybrGreen (A), Syto 9 (B), Syto 64 (C), SyproRed (D) and FM4-64 (E). Color allocation: green = SybrGreen/Syto 9, red = SyproRed/FM4-64. The pyrite surface is shown in reflection mode (= grey).

**Fig 2:**
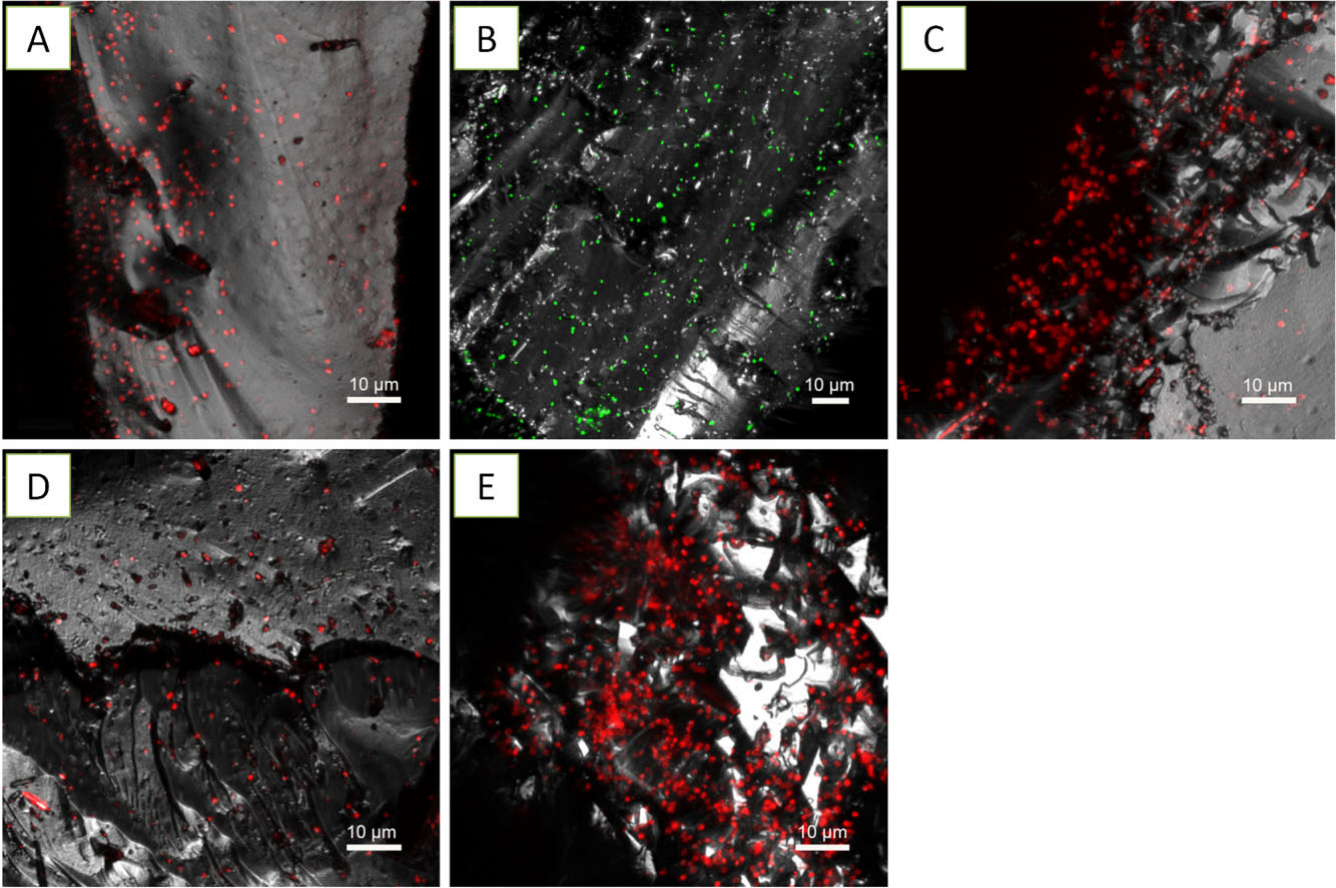
Maximum intensity projections of *Acidianus* sp. DSM 29099 biofilms on pyrite stained by Syto 64 (A), SybrGreen (B) and SyproRed (C). *Sulfolobus metallicus*^T^ biofilms on pyrite stained by Syto 64 (D) and SyproRed (E). Color allocation: green = SybrGreen, red = Syto 64/SyproRed, grey = reflection.

As negative control, the abovementioned dyes including fluoroconjugated lectins were selected randomly to stain sterile pyrite for evaluation of their unspecific binding. Surface structures of sterile, cleaned pyrites showed no unspecific binding of dyes when examined by CLSM (not shown). As shown in Fig. 1A–C, cells of *F. acidiphilum* DSM 28986 attached to pyrite were successfully stained by SybrGreen, Syto 9 and Syto 64. Similarly, cells of *Acidianus* sp. DSM 29099 and *S. metallicus*^T^ were also clearly visualized by staining with these dyes (Fig. 2A, B, D and E). In addition, SyproRed stained cells of *F. acidiphilum* DSM 28986 (Fig. 1D), *Acidianus* sp. DSM 29099 (Fig. 2C) and *S. metallicus*^T^ (Fig. 2E). FM4-64 stained cells of the *Sulfolobales* (not shown) and *F. acidiphilum* DSM 28986 (Fig. 1E). SyproRed and FM4-64 staining gave clear cell-corresponding signals. Therefore, these fluorochromes were used for counter staining in the following tests for cell localization.

In this study, Sypro was used in order to examine archaeal cell surfaces as well as extracellular features. *Ferroplasma*, *Acidiplasma* and *Thermoplasma*, unlike other Archaea, lack a cell wall (Golyshina *et al*., 2000; 2009). It has been shown that the cytoplasmic membrane of *F. acidiphilum* is covered with a thin layer of an amorphous, electron-dense surface matrix (Golyshina and Timmis, 2005). The positive Sypro staining indicates that the thin layer of electron-dense material observed could be a proteinaceous layer, although there is no surface layer (S-layer) characterized in *Ferroplasma*. Another explanation could be that Sypro interacts with membrane proteins. In contrast to *F. acidiphilum*, *Acidianus* sp. DSM 29099 and *S. metallicus*^T^ possess a cell wall, which is mainly composed of S-layer proteins and anchored by their carboxyl-terminal transmembrane domains to the cytoplasmic membrane (Albers and Meyer, 2011). Obviously, S-layer proteins of these two thermophilic archaeal strains were recognized by SyproRed (Fig. 2C and E).

Besides cell visualization, these protein-, lipid- and nucleic acid-specific dyes should also allow the detection of proteins, lipids and DNA as part of the EPS in biofilms (Neu and Lawrence, 2014). In this study, staining of three archaeal strains by abovementioned fluorochromes was mostly restricted to cells, as no smear or diffuse signals around cells were visible (Figs 1 and 2). This indicates that the EPS components including proteins, lipids and eDNA were not present in colloidal fractions or below their detection limit if assessed by means of CLSM. These findings are in good agreement with the EPS analysis by colorimetric methods. The colloidal EPS of *F. acidiphilum* DSM 28986 as well as *Acidianus* sp. DSM 29099 grown on pyrite mainly contained polysaccharides. In contrast, capsular EPS contained both polysaccharides and proteins (R. Y. Zhang, unpublished). In this context, extracellular proteins on cell surface were also stained by SyproRed (Figs 1D, 2C and E). We did not detect eDNA in both cases. It is widely accepted that eDNA has a crucial role in biofilm development and dynamics (Whitchurch *et al*., 2002; Karatan and Watnick, 2009). However, as DNA is a costly molecule for the cell to synthesize, it is reasonable to assume that the chemolithotrophic organisms tested, which obtain little energy by oxidation of pyrite, are not excreting measurable amounts of DNA.

In general, cells of the three species were heterogeneously distributed and developed monolayer biofilms on pyrite surfaces. Large pyrite areas remained uncolonized (∼ 90%). Nevertheless, two levels of spatial organization were observed: cells and small clusters of cells (Figs 1 and 2). These results were confirmed by atomic force microscopy (AFM) combined with epifluorescence microscopy (EFM) (Supporting Information Fig. S1). It must be noted that cell attachment by the strains to pyrite did not occur randomly. Cells of *Acidianus* sp. DSM 29099 and *S. metallicus*^T^ preferentially colonized surface locations with defects (Fig. 2). During the examination of pyrite grains, it became obvious that highly colonized grains exhibited more scratches, microcracks or grooves as compared with the less colonized ones. More pits were observed when pyrite was leached by cells of *Acidianus* sp. DSM 29099 or *S. metallicus*^T^ as compared with *F. acidiphilum* DSM 28986 (Figs 1 and 2). *Acidianus* sp. DSM 29099 and *S. metallicus*^T^, due to their increased growth temperature and their ability to oxidize RISCs arising from pyrite dissolution, have a much higher pyrite leaching capacity than *F. acidiphilum* DSM 28986 (approximately 25 times, Table 2). In a previous report, cells of *Metallosphaera* and *Sulfolobus* spp. did not exhibit any preferential orientation when they attached to pyrite (Etzel *et al*., 2008). This maybe ascribed to the use of different pyrite qualities having different surface properties (e.g. crystallographic orientation).

**Table 2:** Comparison of pyrite leaching activities the strains used after 20 days of cultivation

Strain	Fe total (mg l^−1^)	Fe III/Fe II ratio	Temperature
*Ferroplasma acidiphilum* DSM 28986	255	0.4	37°C
*Sulfolobus metallicus*^T^	6353	6	65°C
*Acidianus* sp. DSM 29099	5913	2.7	65°C

#### *Acidianus* sp. DSM 29099 on elemental sulfur

**Fig 3:**
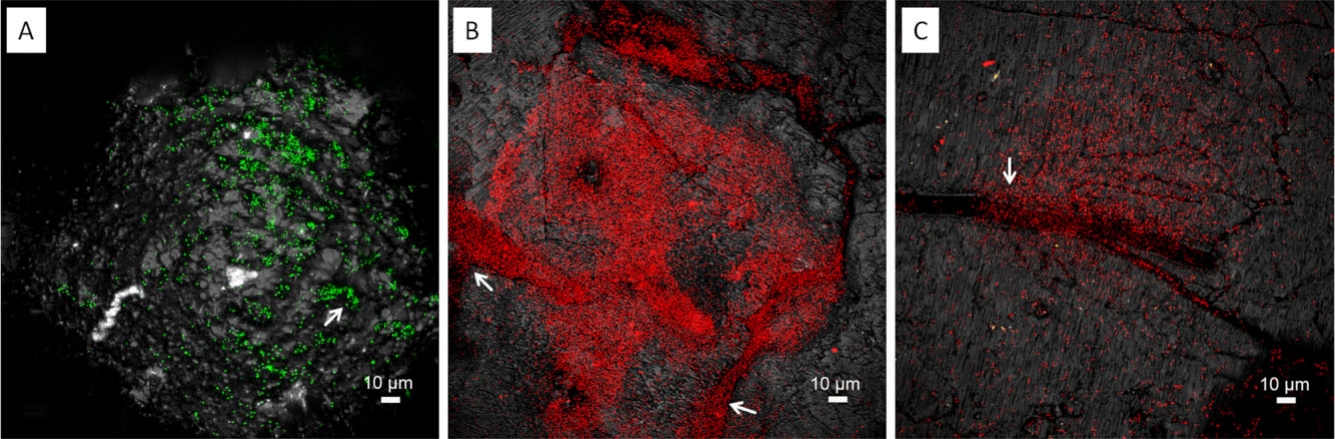
Maximum intensity projections of biofilms from *Acidianus* sp. DSM 29099 on elemental sulfur. Samples were stained by SybrGreen (A), SyproOrange (B) and Syto 64 (C). Color allocation: green = SybrGreen, red = Syto 64/SyproOrange, grey = reflection. Cells formed a thin biofilm in shallow regions, cracks or holes when sulfur prills were used (A). A clear tendency of cells to attach to physical defects on sulfur coupons is also evident (B and C). Arrows show preferential attachment sites with distortions.

The first observation of acidophilic microbes attached to elemental sulfur was described for *Acidithiobacillus thiooxidans* by means of electron microscopy (Schaeffer *et al*., 1963). The attachment of sulfur-oxidizing microbes to sulfur surfaces has been shown to be favoured by the presence of pili, filamentous or glycoglyx materials (Weiss, 1973; Bryant *et al*., 1984; Blais *et al*., 1994). These studies focused mainly on bacteria, and usually, samples were pre-fixed by glutaraldehyde and dehydrated before visualization. By directly applying different stains including SybrGreen, Syto 64 and SyproRed, biofilm cells of *Acidianus* sp. DSM 29099 were clearly visualized on elemental sulfur under fully hydrated conditions, as shown in Fig. 3. Biofilms were heterogeneously distributed and characterized as individual groups of cells, thin but large colonies with up to 50 μm in diameter. Cells formed large aggregates or dense biofilms, in particular, on some sites with cracks and grooves. These cell distribution patterns suggest that adhesion does not occur randomly, and biofilm formation does not proceed uniformly at the sulfur surface. In this case, the presence of cell aggregates suggest that the physical contact of *Acidianus* cells with sulfur is a necessary step for sulfur solubilization, while the upper cells in the aggregates could be oxidizing soluble RISCs.

### FLBA of biofilms on pyrite and elemental sulfur

**Fig 4:**
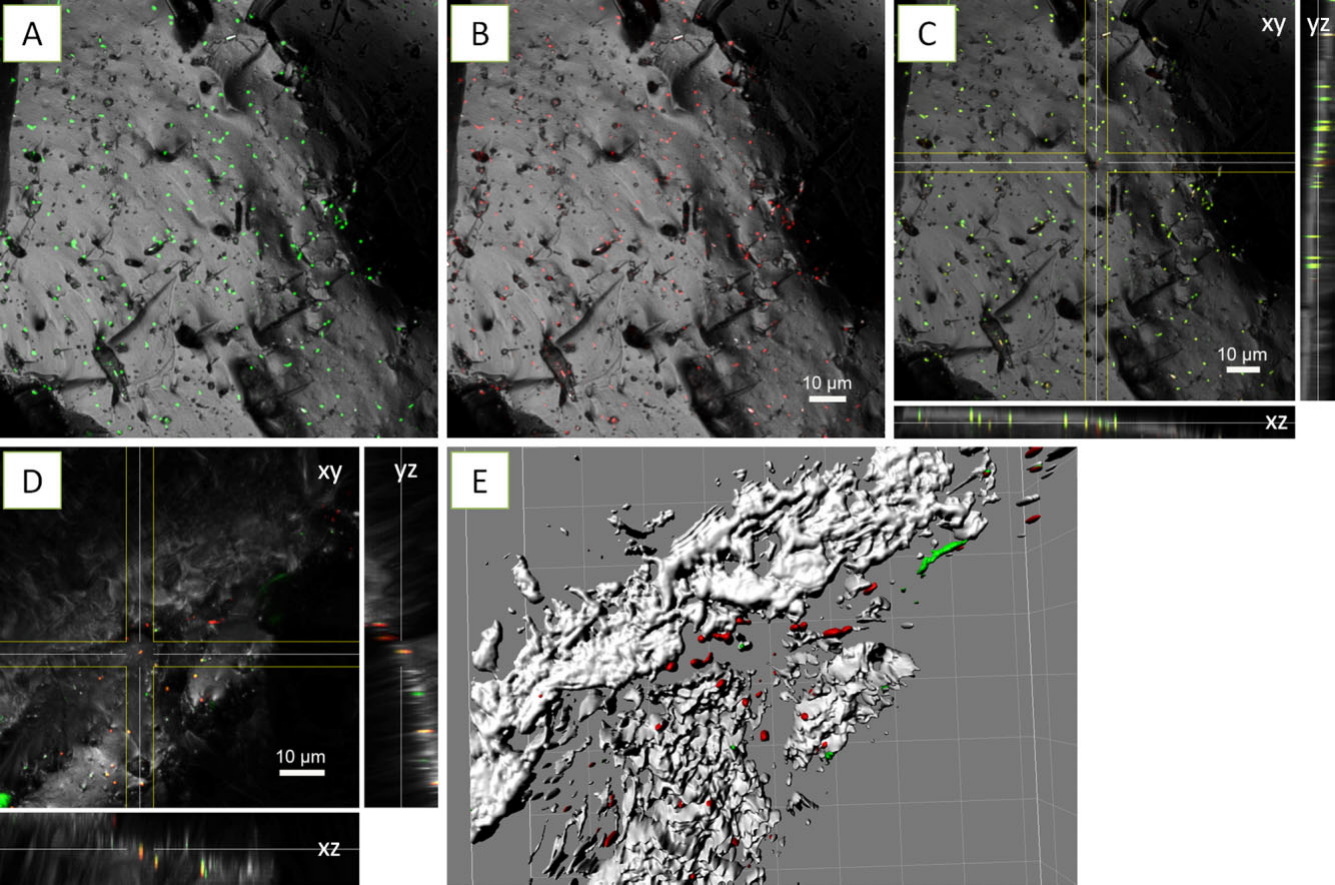
Maximum intensity projections (A, B), XYZ projection (C, D) and isosurface projection (E) of *Acidianus* sp. DSM 29099 biofilms on pyrite stained by Con A (B) and AAL (D, E), and counter stained by and SybrGreen (A) and Syto 64 (D, E). Color allocation: green = SybrGreen/AAL-fluorescein isothiocyanate (FITC), red = Syto 64/Con A-tetramethyl rhodamine isothiocyanate (TRITC), grey = reflection.

The application of FLBA usually includes a screening of all commercially available lectins for probing their reaction with glycoconjugates of (archaeal) biofilms. With the most suitable lectins (for acidophilic archaea), the production of glycoconjugates during biofilm formation may be monitored. In combination with nucleic acid-specific fluorochromes, samples can be analyzed by multichannel CLSM, which has several advantages in analyzing structuring features of hydrated biofilms (Stewart *et al*., 1995; Lawrence *et al*., 1998; Neu and Lawrence, 1999b; 2002). As shown in Table 3, pyrite-grown cells of the three species tested were stainable by 22 (eight for *F. acidiphilum* DSM 28986, eight for *Acidianus* sp. DSM 29099 and 14 for *S. metallicus*^T^) out of 75 lectins. In addition, three binding patterns of lectins to EPS glycoconjugates on pyrite surfaces could be differentiated (Table 3). As indicated in Fig. 4A–C (see also Supporting Information Fig. S2), Con A bound to the cell surface of *Acidianus* sp. DSM 29099. Pyrite surfaces free of cells showed no Con A binding, indicating that Con A only reacted with tightly bound EPS of *Acidianus* sp. DSM 29099.

**Table 3:** Results of lectin binding assays to extracellular glycoconjugates of three archaeal strains on pyrite

Lectins^a^	Specificities	*Ferroplasma acidiphilum* DSM 28986	*Acidianus* sp. DSM 29099	*Sulfolobus metallicus*^T^
AAL	Fuc	+	+	+
		Cell-associated structures	Colloidal	Cell-associated structures
AIA	Gal; GalNAc	+	–	–
			Capsular	
Con A	Man; Glc	+	+	+
		Capsular	Capsular	Capsular
DGL	Man; Glc	+	–	–
		Colloidal		
GNA	Man	–	+	+
			Capsular	Colloidal
GS-I	Gal; GalNAc	–	+	+
			Cell-associated structures	Cell-associated structures
HHA	Man	–	–	+
				Capsular
HPA	GalNAc	+	+	–
		Capsular	Colloidal	
IAA	GalNAc	–	–	+
			Cell-associated structures	
LEA	GlcNAc	–	+	–
			Cell-associated structures	
LPA	Sia	+	–	–
		Colloidal		
MNA-G	Gal	–	–	+
				Capsular
MPA	GalNAc	–	+	–
			Colloidal	
PA-I	Gal	+	–	–
		Capsular		
PHA-E	Man	+	–	+
		Colloidal		Capsular
PHA-L	GalNAc	–	–	+
				Capsular
PMA	Man	–	+	–
			Capsular	
PSA	Man	–	–	+
				Capsular
SJA	GalNAc	–	–	+
				Colloidal
STA	GluNAc			Capsular
VVA	GalNAc	–	–	+
				Cell-associated structures
WFA	GalNAc	–	–	+
				Capsular

a The details of all lectins used in this study are shown in Supporting Information Table S1. Staining and visualization procedures are described in the section Experimental procedures.

+, lectin binding; −, no binding; AIA, *Artocarpus integrifolia* agglutinin; DGL, *Dioclea grandiflora lectin*; GNA, *Galanthus nivalis* agglutinin; HHA, Amaryllis lectin; HPA, *Helix pomatia* agglutinin; IAA, *Iberis amara* agglutinin; LEA, *Lycopersicon esculentum* agglutinin; MNA-G, Morniga G; MPA, *Maclura pomifera* agglutinin; PA-I, *Pseudomonas aeruginosa* lectin I; PHA-E, *Phaseolus vulgaris* agglutinin E; PHA-L, *Phaseolus vulgaris* agglutinin L; PMA, *Polygonatum multiflorum* agglutinin; PSA, *Pisum sativum* agglutinin; SJA, *Sophora japonica* agglutinin; STA, *Solanum tuberosum* agglutinin; VVA, *Vicia villosa* agglutinin; WFA, *Wisteria floribunda* agglutinin.

**Fig 5:**
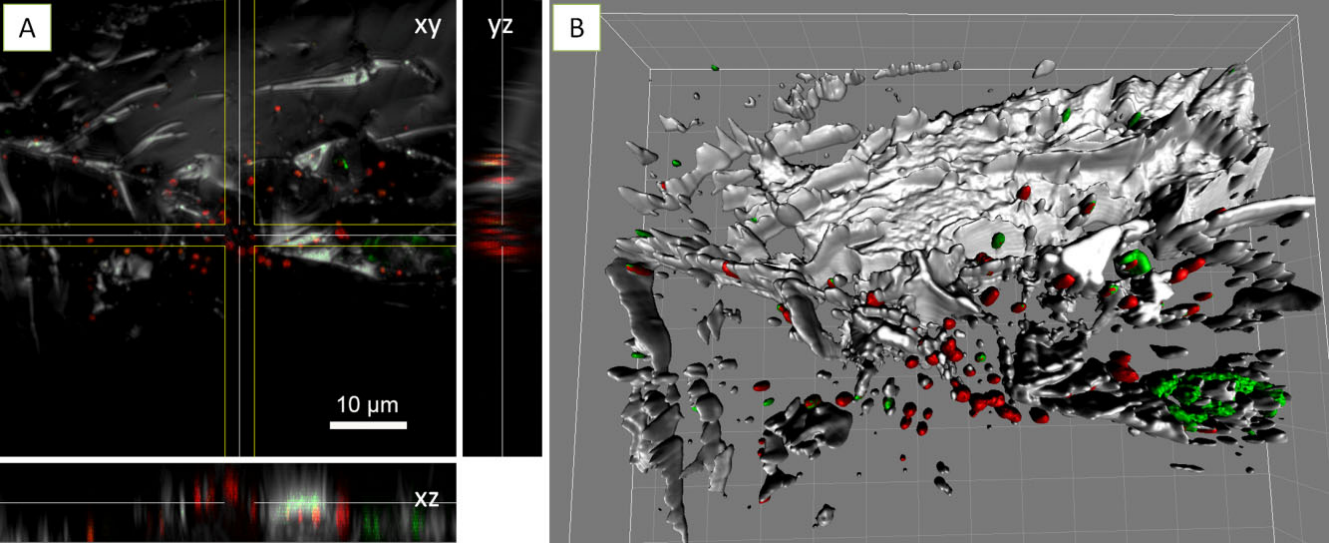
XYZ projection (A) and isosurface projection (B) of *F. acidiphilum* DSM 28986 biofilms on pyrite stained by LPA-fluorescein isothiocyanate (FITC) and counter stained by FM4-64. Color allocation: green = LPA-FITC, red = FM4-64, grey = reflection. Grid size in B = 10 μm.

The lectins *Aleuria aurantia* lectin (AAL) and *Limulus polyphemus* agglutinin (LPA) stained biofilm cells of *Acidianus* sp. DSM 29099 (Fig. 4D and E) and *F. acidiphilum* DSM 28986 as well as their surrounding sites (Fig. 5). Unlike the binding of Con A to cells on pyrite surface, these lectins also bound to cell-free EPS on pyrite surfaces. This ‘colloidal’ EPS binding pattern may allow us to speculate that capsular EPS are probably involved in the initial attachment, and consequently, more EPS were produced in form of colloidal EPS by cells building biofilms on pyrite. Thus, as ocurring in bioleaching bacteria, large areas of the surface may actually be devoid of cells but may be covered by ‘colloidal’ EPS (Sand *et al*., 2001). In this context, it has been reported that *T. litoralis* excretes exopolysaccharides into the growth medium and that these may cause a conditioning layer on surfaces (Rinker and Kelly, 1996).

**Fig 6:**
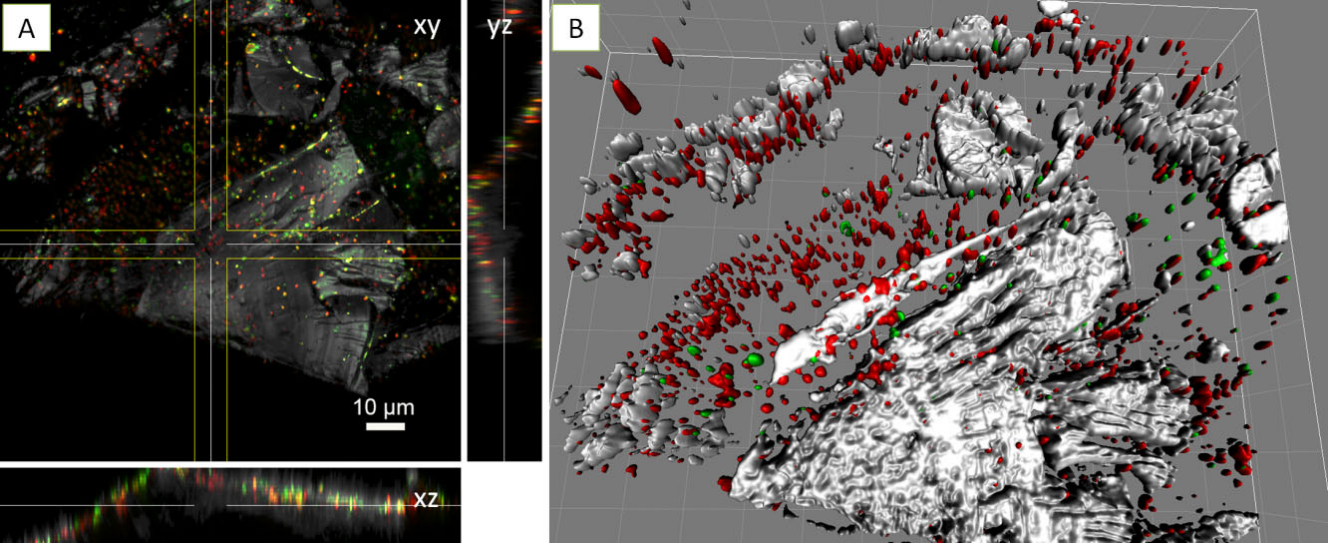
XYZ projection (A) and isosurface projection (B) of *Acidianus* sp. DSM 29099 biofilms on pyrite stained by GS-I-fluorescein isothiocyanate (FITC) and counter stained by Syto64. Color allocation: green = GS-I-FITC, red = Syto 64, grey = reflection. Grid size in B = 10 μm.

Fig. 6 shows that the cell surfaces of *Acidianus* sp. DSM 29099 and *S. metallicus*^T^ clearly reacted with the lectin *Griffonia simplicifolia* lectin (GS-I). Within stained biofilms, GS-I signals mostly covered the cells but also filled the space between cells and pyrite surfaces. This cell-associated binding pattern indicates the potential complexity of biofilm structures. It can be concluded that the lectins showing cell-associated binding patterns reacted with relatively more biofilm components than the ones present just in capsular or colloidal EPS.

#### Glycoconjugates produced on pyrite

The glycoconjugates present in biofilms of the three archaeal species on pyrite are listed in Table 3. Based on lectin specificity, fucose, glucose, N-acetylgalactosamine (GalNAc), galactose and mannose were found in the three tested species.

*Acidianus* sp. DSM 29099 and *S. metallicus*^T^ biofilms on pyrite were shown to possess similar glycoconjugates including fucose, GlcNAc, GalNAc, galactose, mannose and glucose. Both GalNAc and GlcNAc are present in archaeal cell walls. As *Ferroplasma* lacks a cell wall, it is not surprising that we did not detect GlcNAc in these archaea. The binding of the lectin LPA to biofilm cells strongly suggest *F. acidiphilum* DSM 28986 to possess sialic acid residues. Sialic acids are a family of about 50 derivatives of *N*-acetyl or *N*-glycolyl neuraminic acids. They typically occupy the distal end of glycan chains, which makes them suitable for interaction with other cells or with environmental constituents and interfaces. Sialic acids are mainly found in animals and their pathogens, and in certain bacteria (Angata and Varki, 2002; Vimr *et al*., 2004). They are important components of glycoproteins and glycolipids in animal cell membranes. Studies concerning the presence of sialic acid in archaea are rare (Angata and Varki, 2002; Lewis *et al*., 2009). By applying reverse-phase high-performance liquid chromatography combined with a fluorescent labelling method using 1,2-diamino-4,5-methylenedioxybenzene dihydrochloride to label the sialic acids (Hara *et al*., 1989), we were able to confirm the presence of sialic acids in cells of *F. acidiphilum* DSM 28986 (R. Y. Zhang & V. Blanchard, unpublished). Additionally, planktonic cells of this microorganism were not stained by LPA. As sialic acid residues are mostly present in biofilms of *F. acidiphilum* DSM 28986 grown on pyrite, it can be assumed that these residues play an important role in attachment and presumably also in the biologically accelerated oxidation of pyrite. Consequently, these results indicate that acidophilic leaching archaea might use different surface compounds (i.e. sialic acids in case of *F. acidiphilum*) for mediating cell–mineral interactions compared with bacterial ones. These are considered to be established by uronic acids complexing iron(III) ions, which are mediating cell attachment by electrostatic interactions and increase the concentration of the pyrite oxidizing agent iron(III) ions (Sand *et al*., 2001).

A few lectins including Con A, GS-I, GS-II and WGA have been applied to stain and visualize EPS of *Sulfolobus* spp. WGA was first reported to stain *S. acidocaldarius* and *Sulfolobus shibatae* on polycarbonate membrane filters (Fife *et al*., 2000). In our assays, this lectin did not bind to any of the strains tested. The lectins Con A, GS-I and GS-II have been used in studies on *S. acidocaldarius*, *S. solfataricus* and *S. tokodaii*, including some *S. solfataricus* mutants defective in biofilm formation and production of cell surface appendages (Koerdt *et al*., 2010; 2012; Zolghadr *et al*., 2010; Henche *et al*., 2012). The reaction of these lectins with *Acidianus* sp. DSM 29099 and *S. metallicus*^T^ (Tables 3 and 4) strongly indicates that especially Con A and GS-I can be used to monitor biofilm formation of *Sulfolobales*. However, it is important to remark that the abovementioned species are normally grown in complex media (e.g. 0.1% tryptone or 0.2% maltose), while in our experiments, we have used pyrite or elemental sulfur (in case of *Acidianus* sp. DSM 29099) as energy sources. Under these conditions, most of the carbon for biosynthesis must be fixed from CO_2_. Control experiments showed no significant cell growth of *Acidianus* sp. DSM 29099 with 0.02% yeast extract as sole energy source (not shown). As we focused on biofilm formation on pyrite or sulfur surfaces, we cannot rule out that under presence of sufficient amounts of organic carbon, *Acidianus* sp. DSM 29099 may build structurally more complex biofims as described for other *Sulfolobales* (Koerdt *et al*., 2010).

**Table 4:** Results of lectin binding assays to extracellular glycoconjugates of *Acidianus* sp. DSM 29099 on elemental sulfur

Lectins	Binding pattern	Lectins	Binding pattern
AAA	Capsular	AAL	Capsular/cell-associated structures
Con A	Capsular/cell-associated structures	DBA	Capsular
ECA	Colloidal	EEA	Capsular
GHA	Capsular	GS-I	Colloidal/cell-associated structures
HHA	Colloidal	HMA	Colloidal
IAA	Colloidal	IRA	Capsular
LAL	Capsular	LBA	Capsular
LcH	Capsular	MOA	Colloidal
PNA	Capsular	PSA	Capsular
SJA	Capsular	TL	Capsular
VGA	Capsular		

DBA, *Dolichos biflorus* agglutinin; ECA, *Erythrina cristagalli* agglutinin; EEA, *Euonymus europaeus* agglutinin; GHA, *Glechoma hederacea* agglutinin; HMA, *Homarus americanus* agglutinin; IRA, *Iris hybrid* agglutinin; LAL, *Laburnum anagyroides lectin*; LBA, *Phaseolus lunatus* agglutinin; LcH, *Lens culinaris* haemagglutinin; MOA, *Marasmium oreades* agglutinin; PNA, Peanut agglutinin; TL,*Tulipa* sp. agglutinin; VGA, *Vicia graminea* agglutinin.

The lectins AAL and Con A stained cells of the three species used in this study, which is consistent with previous reports that these two lectins have the potential to stain various kinds of biofilms (Neu and Lawrence, 2002; Neu *et al*., 2002; Strathmann *et al*., 2002; Staudt *et al*., 2003; Bellenberg *et al*., 2012). The monomers glucose, mannose and fucose were also found in EPS fractions of *Acidithiobacillus ferrooxidans* (Gehrke *et al*., 1998). Thus, leaching bacteria and archaea may have similarities in their EPS composition.

#### Glycoconjugates produced on elemental sulfur

**Fig 7:**
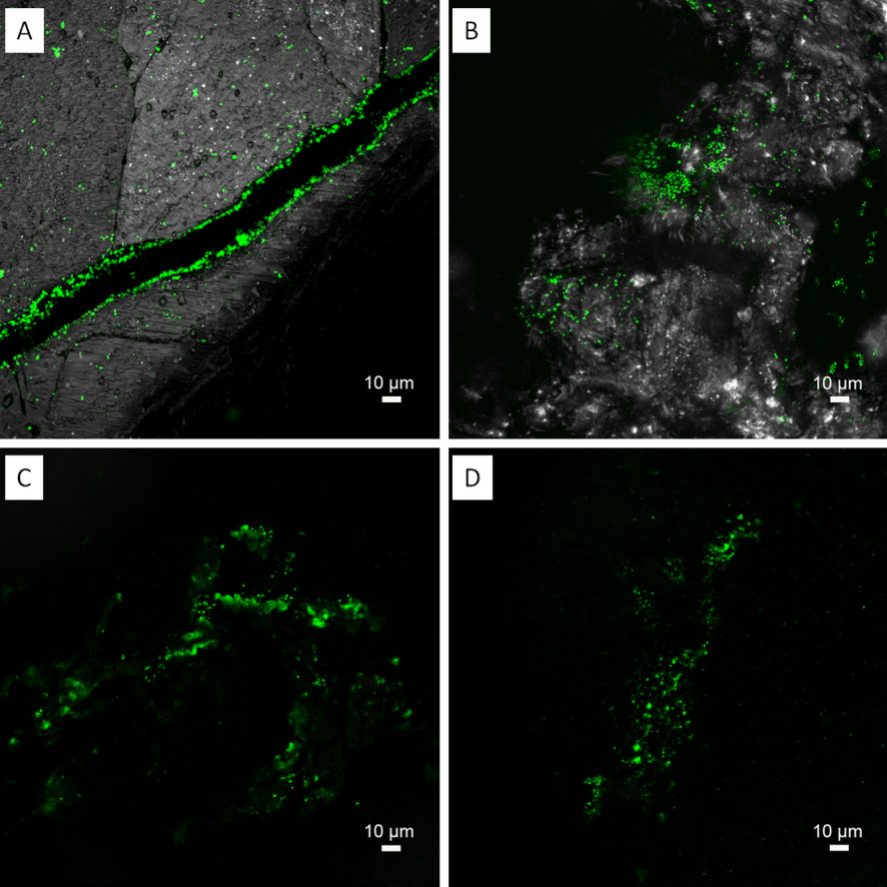
Maximum intensity projections of biofilms from *Acidianus* sp. DSM 29099 on elemental sulfur. Samples were stained by lectins AAL-Alexa488 (A), Peanut agglutinin (PNA)-fluorescein isothiocyanate (FITC) (B), *Erythrina cristagalli* agglutinin (ECA)-FITC (C) and GS-I (D). Two distinguished lectin binding patterns became visible, tightly bound “capsular” EPS staining (A and B) and loosely bound “colloidal” (C and D). Color allocation: green = lectins, grey = reflection.

Twenty-one lectins were shown to bind EPS glycoconjugates of *Acidianus* sp. DSM 29099 (Table 4). In addition, two major binding patterns became evident. Five lectins showed signals covering cells and an extended area around them, indicating their binding to colloidal or loosely bound EPS. In contrast, 16 lectins showed a ‘capsular binding pattern’, in which signals were only restricted to cells (Fig. 7 and Supporting Information Fig. S2). Among these 21 lectins, only AAL, Con A and GS-I stained EPS glycoconjugates of *Acidianus* sp. DSM 29099 on pyrite (Tables 3 and 4). It indicates that the glycoconjugates, which these three lectins recognized (i.e. glucose, mannose, GalNAc and galactose), are common components of *Acidianus* sp. DSM 29099 attached on pyrite and sulfur surfaces. From another point of view, *Acidianus* cells may express different glycoconjugates which correlate with their energy sources. In addition, extracellular proteins of *Sulfolobales* are highly glycosylated. N-glycans usually consist of glucose, mannose, GalNAc and GlcNAc. The high glycosylation density in S-layers could represent an adaptation of these organisms to the high temperature and acidic environment that they naturally encounter (Meyer and Albers, 2013). It has been shown that the loss of one terminal hexose of the N-glycan has effects on cell growth and motility (Jarrell *et al*., 2014). We suggest that some of the lectins bound also to the N-glycans from the S-layer, which are probably differentially expressed between pyrite and sulfur-grown cells in biofilms of *Acidianus* sp. DSM 29099.

## Conclusions

We studied a library of lectins for their potential to visualize and characterize glycoconjugates in acidophilic archaeal biofilms. The lectin binding tests provided the first hint for the distribution of glycoconjugates that are involved in biofilm formation on pyrite as well as on elemental sulfur. By FLBA, the glycoconjugates in acidophilic archaeal biofilms were characterized as polymers containing sugar moieties like glucose, galactose, mannose, GlcNAc, GalNAc, sialic acid and fucose. Twenty-two lectins were shown to be useful for the study of EPS glycoconjugates of acidophilic archaea during pyrite dissolution. These lectins may be used in future studies for assessment of interactions between various members of microbial bioleaching communities, especially in order to elucidate the role of archaea in detail. In addition, lectins which are species or strain specific (e.g. LPA staining *F. acidiphilum*) may be used as probes to differentiate a target archaeon from others in multi-species biofilm studies.

## Experimental procedures

### Archaeal strains and cultivation

*Ferroplasma acidiphilum* DSM 28986 = JCM 30201 (former BRGM4) was isolated from a stirred tank reactor (Bryan *et al*., 2009). *Sulfolobus metallicus* DSM 6482^T^ was purchased from DSMZ. Strain *Acidianus* sp. DSM 29099 = JCM 30227, an iron- and sulfur-oxidizer, was isolated from a hot spring at Copahue Volcano, Neuquén, Argentina (R. Y. Zhang & W. Sand, unpublished). All strains were cultivated in MAC medium (Mackintosh, 1978) containing 0.02% yeast extract with an initial pH 1.8 for *F. acidiphilum* DSM 28986 or pH 2.5 for *S. metallicus*^T^ and *Acidianus* sp. DSM 29099 respectively. *Ferroplasma acidiphilum* DSM 28986 was grown at 37°C with 4 g l^−1^ iron(II) ions. *Sulfolobus metallicus*^T^ and *Acidianus* sp. DSM 29099 were grown at 65°C with 10 g l^−1^ elemental sulfur.

### Substratum, biofilm formation and pyrite leaching

Pyrite grains with a size of 200–500 μm were selected after grinding and sieving of pyrite cubes from Rioja (Spain). They were cleaned and sterilized as described (Schippers *et al*., 1996; Schippers and Sand, 1999). For cell attachment assays, 20 g of sterile pyrite grains were incubated with pure cultures of each strain in 300 ml Mac (initial cell concentration 10^8^ cells/ml) to allow biofilm development and pyrite dissolution. Iron ions were quantified by the phenanthroline method (Tamura *et al*., 1974).

Sulfur powder (Roth, Germany) was molten and poured into deionized water with agitation. Sulfur prills with a diameter of 1–3 mm were formed due to rapid cooling. A plate covered with aluminium was used to get a sulfur layer after solidification. Sulfur coupons with a size of approximately 0.5 cm × 0.5 cm × 2 mm were obtained by breaking the sulfur layer. Both sulfur prills and coupons were sterilized at 110°C for 90 min.

### Staining

Cell distribution was observed after nucleic acid staining with Syto 9, Syto 64 or SybrGreen. In addition, SyproRed, binding to cellular proteins, or FM4-64, binding to lipid-rich domains (Bolte *et al*., 2004), were used for counter staining. For glycoconjugate staining, biofilms of each cell population were assayed with lectins conjugated with fluorescein isothiocyanate, Alexa 488 or tetramethyl rhodamine isothiocyanate (Supporting Information Table S1). Briefly, samples were washed with filter-sterilized tap water and incubated with 0.1 mg ml^−1^ lectins for 20 min at room temperature. Afterwards, stained samples were washed three times with filter-sterilized tap water in order to remove unbound lectins. Direct light exposure was avoided. Counter staining was done in a coverwell chamber of 20 mm in diameter with 0.5 mm spacer (Invitrogen). Counter-stained samples were directly observed using CLSM without any further treatment.

### CLSM

Examination of stained biofilms was performed by CLSM using a TCS SP5X (Leica, Heidelberg, Germany), controlled by the lasaf 2.4.1 build 6384. The system was equipped with an upright microscope and a super continuum light source (470–670 nm) as well as a 405 nm laser diode. Images were collected with a 63 × water immersion lens with a numerical aperture (NA) of 1.2 and a 63 × water immersible lens with an NA of 0.9. The details of fluorescent dyes along with excitation and emission filters are shown in Table 1. CLSM data sets were recorded in sequential mode in order to avoid cross talk of the fluorochromes between two different channels. Surface topography and texture of the pyrite as well as of the elemental sulfur surface were recorded by using the CLSM in reflection mode.

### AFM and EFM

Pyrite slices were rinsed with sterile MAC medium and deionized water. Cells attached to pyrite coupons and their EPS were stained by Syto 9 and by fluorescently labelled Con A. Stained samples were dried at room temperature and visualized by EFM (Zeiss, Germany) combined with AFM (BioMaterial™Workstation, JPK Instruments) for the investigation of cell morphology and distribution of cells on the surfaces of pyrite coupons (Zhang *et al*., 2014).

### Digital image analysis

Fluorescence images were analyzed using an extended version of software ImageJ (Abràmoff *et al*., 2004). Maximum intensity and XYZ projections of three-dimensional data sets were produced with the software imaris version 7.3.1 (Bitplane AG, Zurich, Switzerland).

## Supporting Information

**Fig. S1.** Biofilm cells of *Acidianus* sp. DSM 29099 visualized by atomic force microscope (AFM) combined with epifluorescence microscope (EFM), exhibiting preferential attack on the pyrite lattice along planes. A and B show EFM images of *Acidianus* sp. DSM 29099 biofilms stained by Syto 9 (green) and TRITC-conjugated Con A (red), respectively. C shows AFM scanning corresponding to EFM (A and B). White arrows show a cell cluster. Bars represent 10 μm. **Fig. S2.** Biofilm cells of *Acidianus* sp. DSM 29099. A, cells grown on pyrite and stained by TRITC-conjugated Con A (red) and Syto 9 (green), respectively. B, cells grown on elemental sulfur and stained by FITC-conjugated Con A (green). Con A stained cell surfaces and gave a clear ‘capsular binding’ pattern. Arrows show cell surfaces. Bars represent 5 μm. **Table S1.** List of fluorescent labeled lectins used for staining of archaeal biofilms and their binding target.
