# Supplementary material for: Use of lectins to in situ visualize glycoconjugates of extracellular polymeric substances in acidophilic archaeal biofilms
Source: Microb Biotechnol. 2014 Dec 9;8(3):448–61. doi: 10.1111/1751-7915.12188 (PMC4408177; doi:10.1111/1751-7915.12188)
Supplement: Supplementary file 1 [file mbt20008-0448-sd1.pdf]

Supplementary Material

Use of lectins to in situ visualize glycoconjugates of extracellular polymeric substances in acidophilic archaeal biofilms

R.Y. Zhang[1], T. R. Neu[2], S. Bellenberg[1], U. Kuhlicke[2], W. Sand[1] and M. Vera[1*]

[1]*Universität Duisburg – Essen, Biofilm Centre, Aquatische Biotechnologie, Universitätsstraße 5, 45141, Essen, Germany*

[2]*Helmholtz Centre for Environmental Research-UFZ, Department of River Ecology, Brueckstrasse 3A ,39114, Magdeburg, Germany*

**Corresponding Author*

E-mail: mario.vera@uni-due.de

Tel: + 49 0201/183 7080

Fax: + 49 0201/183 7088

Running title: A lectin study of archaeal glycoconjugates during bioleaching

Keywords Bioleaching · Biofilm · Archaea · Lectin

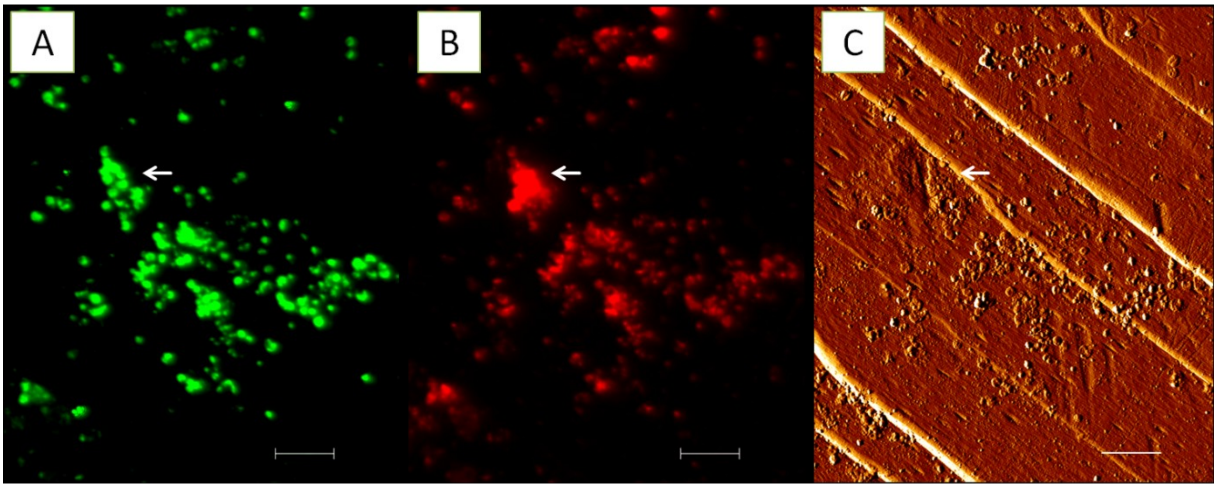

Supplementary Fig. S1. Biofilm cells of *Acidianus* sp. DSM 29099 visualized by atomic force microscope (AFM) combined with epifluorescence microscope (EFM), exhibiting preferential attack on the pyrite lattice along planes. A and B show EFM images of *Acidianus* sp. DSM 29099 biofilms stained by Syto 9 (green) and TRITC-conjugated Con A (red), respectively. C shows AFM scanning corresponding to EFM (A and B). White arrows show a cell cluster. Bars represent 10 µm. For details of the AFM & EFM visualization procedure, see the section Experimental procedures and Mangold *et al*., 2008.

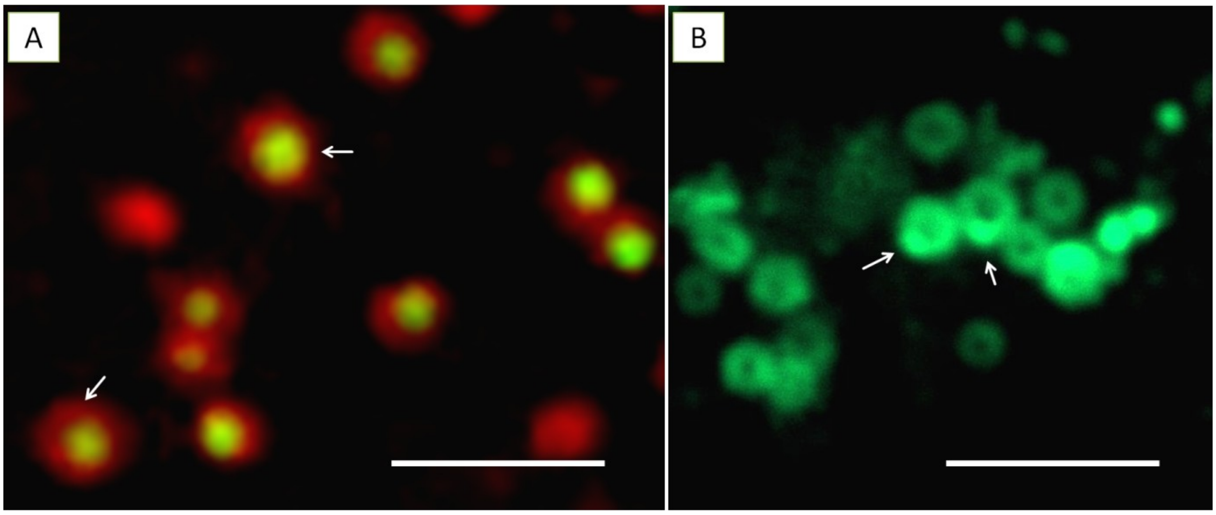

Supplementary Fig. S2. Biofilm cells of *Acidianus* sp. DSM 29099. A, cells grown on pyrite and stained by TRITC-conjugated Con A (red) and Syto 9 (green), respectively. B, cells grown on elemental sulfur and stained by FITC-conjugated Con A (green). Con A stained cell surfaces and gave a clear 'capsular binding' pattern. Arrows show cell surfaces. Bars represent 5 µm.

Supplementary Table S1. List of fluorescent labeled lectins used for staining of archaeal biofilms and their binding target

Name and abbreviation*	Source	Specificity	Lectin group[#]
FITC labeled lectins			
Anguilla anguilla agglutinin, AAA	Eel serum (*Anguilla anguilla*)	Fuc	Animal
Agaricus bisporus agglutinin, ABA/ABL	Edible mushroom (*Agaricus bisporus*)	GalNAc; Gal	Fungi
Amaranthin, ACA/ACL	*Amaranthus caudatus*	GalNAc	Plant
Artocarpus integrifolia agglutinin, AIA	*Artocarpus integrifolia*	GalNAc; Gal	Plant
Arum maculatum agglutinin, AMA	*Arum maculatum*	Man	Plant
Aegopodium podagraria lectin, APP	*Aegopodium podagraria*	GalNAc	Plant
Allium sativum agglutinin, ASA	Garlic (*Allium sativum*)	Man	Plant
Bryonia dioica agglutinin, BDA	*Bryonia dioica*	GalNAc	Plant
Bauhinia purpurea agglutinin, BPA	*Bauhinia purpurea*	GalNAc	Plant
Colchicum autumnale lectin, CA	Meadow saffron (*Colchicum autumnale*)	Lac; GalNAc; Gal	Plant
Caragana aborescens agglutinin, CAA	*Caragana arborescens*	GalNAc	Plant
Calystegia sepium lectin, Calsepa	Hedge bindweed (*Calystegia sepium*)	Man/Mal	Plant
Concanavalin A, Con A	Jack bean (*Canavalla ensiformis*)	Glc, Man	Plant
Cicer arietinum agglutinin, CPA	Chick pea (*Cicer arietinum*)	Complex	Plant
Cytisus scoparius agglutinin,	Scotch broom (*Cytisus scoparius*)	GalNAc; Gal	Plant

CSA			
Dolichos biflorus agglutinin, DBA	*Dolichos biflorus*	GalNAc	Plant
Dioclea grandiflora lectin, DGL	*Dioclea grandiflora*	Glc, Man	Plant
Datura stramonium agglutinin, DSA	*Datura stramonium*	GlcNAc	Plant
Erythrina cristagalli agglutinin, ECA	*Erythrina cristagalli*	GalNAc; Gal	Plant
Euonymus europaeus agglutinin, EEA	*Euonymus europaeus*	Gal	Plant
Glechoma hederacea agglutinin, GHA	Ivy (*Glechoma lederacea*)	GalNAc	Plant
Galanthus nivalis agglutinin, GNA	Snowdrop (*Galanthus nivalis*)	Man	Plant
Griffonia simplicifolia lectin, GS-I	*Griffonia simplicifolia*	GalNAc; Gal	Plant
Helix aspersa agglutinin, HAA	Snail (*Helix aspersa*)	GalNAc	Animal
Amaryllis lectin, HHA	Amaryllis (*Hippeastrum hybrid*)	Man	Plant
Homarus americanus agglutinin, HMA	Lobster (*Homarus americanus*)	Sia	Animal
Helix pomatia agglutinin, HPA	Edible snail (*Helix pomatia*)	GalNAc	Animal
Iris hybrid agglutinin, IRA	Dutch iris (*Iris hybrid*)	GalNAc	Plant
Laburnum alpinum agglutinin, LAA	*Laburnum alpinum*	Gal	Plant
Laburnum anagyroides lectin, LAL	*Laburnum anagyroides*	Fuc	Plant
Phaseolus lunatus agglutinin, LBA	Lima bean (*Phaseolus lunatus*)	GalNAc	Plant
Lens culinaris haemagglutinin, LcH	Lentil (*Lens culinaris*)	Glc, Man	Plant

Lycopersicon esculentum agglutinin, LEA	Tomato (*Lycopersicon esculentum agglutinin*)	GlcNAc	Plant
Limax flavus agglutinin, LFA	Slug (*Limax flavus*)	Sia	Animal
Tetragonolobus purpurea lectin, Lotus	*Tetragonolobus purpurea*	Fuc	Plant
Limulus polyphemus agglutinin, LPA	Horseshoe crab (*Limulus polyphemus*)	Sia	Animal
Maackia amurensis agglutinin, MAA	*Maackia amurensis*	Sia	Plant
Morniga G, MNA-G	*Morus nigra*	Gal	Plant
Marasmium oreades agglutinin, MOA	Mushroom (*Marasmium oreades*)	Gal	Fungi
Maclura pomifera agglutinin, MPA	*Maclura pomifera*	GalNAc	Plant
Narcissus pseudonarcissus agglutinin, NPA	*Narcissus pseudonarcissus*	Man	Plant
Phaseolus vulgaris agglutinin E, PHA-E	*Phaseolus vulgaris*	Man	Plant
Phaseolus vulgaris agglutinin E, PHA-L	*Phaseolus vulgaris*	GalNAc	Plant
Polygonatum multiflorum agglutinin, PMA	*Polygonatum multiflorum*	Man	Plant
Peanut agglutinin, PNA	Peanut (*Arachis hypogea*)	Gal	Plant
Pisum sativum agglutinin, PSA	*Pisum sativum*	Man	Plant
Polyporus squamosus lectin, PSL	Polypore mushroom (*Polyporus squamosus*)	Sia	Fungi
Psophocarpus tetragonolobus agglutinin, PTA	*Psophocarpus tetragonolobus*	GalNAc	Plant
Phytolacca americana agglutinin, PWA	Pokeweed (*Phytolacca americana*)	GlcNAc	Plant
Robinia pseudoaccacia	Black locust (*Robinia*	GalNAc	Plant

agglutinin, RPA	*pseudacacia*)		
Soybean agglutinin, SBA	Soybean (*Glycine max*)	GalNAc; Gal	Plant
Sophora japonica agglutinin, SJA	Japanese pagoda tree (*Sophora japonica*)	GalNAc	Plant
Sambucus nigra agglutinin, SNA	Elderberry (*Sambucus nigra*)	GalNAc; Gal	Plant
Solanum tuberosum agglutinin, STA	*Solanum tuberosum*	GlcNAc	Plant
Trichosanthes kirilowii agglutinin, TKA	*Trichosanthes kirilowii*	Gal	Plant
Tulipa sp. agglutinin, TL	*Tulipa* sp.	GalNAc	Plant
Urtica dioica agglutinin, UDA	*Urtica dioica*	GalNAc	Plant
Ulex europaeus I, UEA I	Furze gorse (*Ulex europaeus*)	Fuc	Plant
Vicia faba agglutinin, VFA	*Vicia faba*	Glc	Plant
Vicia graminea agglutinin, VGA	*Vicia graminea*	GlcNAc	Plant
Vigna radiata agglutinin, VRA	*Vigna radiata*	Gal	Plant
Vicia villosa agglutinin, VVA	*Vicia villosa*	GalNAc	Plant
Wisteria floribunda agglutinin, WFA	*Wisteria floribunda*	GalNAc	Plant
Wheat germ agglutinin, WGA	*Triticum vulgaris*	GlcNAc	Plant
Alexa488 labeled lectins			
Aleuria aurantia lectin, AAL	*Aleuria aurantia*	Fuc	Fungi
Cancer antennarius agglutinin, CCA	Marine crab (*Cancer antennarius*)	Sia	Animal
Codium fragile lectin, Co	*Codium fragile*	GalNAc	Alga
Erythrina corallodendron lectin, Ecor	*Erythrina corallodendron*	Gal	Plant
Homarus americanus agglutinin,	Lobster (*Homarus americanus*)	Sia	Animal
Iberis amara agglutinin, IAA	*Iberis amara*	GalNAc	Plant

Mangifera indica agglutinin, MIA	*Mangifera indica*	ND	Plant
Perseau americana agglutinin, PAA	*Perseau americana*	GlcNAc	Plant
Pseudomonas aeruginosa lectin I, PA-I	*Pseudomonas aeruginosa*	Gal	Bacteria
Ptilota plumosa agglutinin, PPA	*Ptilota plumosa*	Gal	Alga
Trifolium repens agglutinin, RTA	*Trifolium repens*	GlcA	Plant
Tritrichomonas mobilensis lectin, TML	*Tritrichomonas mobilensis*	Sia	Bacteria
Vicia graminea agglutinin, VGA	*Vicia graminea*	GlcNAc	Plant
TRITC labeled lectin			
Con A	Jack bean (*Canavalla ensiformis*)	Glc, Man	Plant

*Man=Mannose, Fuc=Fucose, Glc=Glucose, GlcA=Glucuronic acid, Gal=Galactose, GalNAc=*N*-acetylgalactosamine, GlcNAc=*N*-acetylglucosamine, ND=Not determined, Sia=Sialic acid, Lac=Lactose, Mal=Maltose

#For details of the lectin classification, see Doyle and Slifkin, 1994 and Van Damme *et al*., 1998.

References

Doyle, R.J., and Slifkin, M. (1994) *Lectin-microorganism interactions*. New York, USA: Marcel Dekker.

Mangold, S., Harneit, K., Rohwerder, T., Claus, G., and Sand, W. (2008) Novel combination of atomic force microscopy and epifluorescence microscopy for visualization of leaching bacteria on pyrite. *Appl Environ Microbiol* **74:** 410-415.

Van Damme, E.J., Peumans, W.J., Pusztai, A., and Bardocz, S. (1998) *Handbook of plant lectins: properties and biomedical applications*. Chichester; New York: John Wiley & Sons.
